# Characterization of a subgroup of non-type 2 asthma with cow’s milk hypersensitivity in young subjects

**DOI:** 10.1186/s13601-019-0250-2

**Published:** 2019-02-22

**Authors:** N. Tsolakis, L. Nordvall, C. Janson, N. Rydell, A. Malinovschi, K. Alving

**Affiliations:** 1 Department of Women’s and Children’s Health, Uppsala University Hospital, Uppsala University, 751 85 Uppsala, Sweden; 2 Department of Medical Sciences, Clinical Physiology, Uppsala University, Uppsala, Sweden; 3 Thermo Fisher Scientific, ImmunoDiagnostics, Uppsala, Sweden

## Abstract

**Background:**

Asthma with atopy is often characterized by type 2 inflammation but less progress has been made in defining non-type 2 asthma. We have previously identified a subgroup of young non-atopic asthmatics with perceived food hypersensitivity and poor asthma control.

**Objective:**

Our aim was to further characterize this subgroup of non-type 2 asthmatics, including the use of a broad panel of inflammation-related proteins.

**Methods:**

Sex- and age-matched subjects (10–35 years old) were divided into three groups with regard to history of asthma and atopy: non-atopic asthmatics with perceived cow’s milk hypersensitivity but with IgE antibodies < 0.35 kU_A_/L (NAA; n = 24), non-atopic controls with IgE < 0.35 kU_A_/L (NAC; n = 24), and atopic asthmatics with IgE ≥ 0.35 kU_A_/L (AA; n = 29). Serum or plasma were analysed using the multi-allergen tests Phadiatop and fx5 (ImmunoCAP), a multiplex immunoassay comprising 92 inflammation-related proteins (Proseek Inflammation), and an ELISA for human neutrophil lipocalin (S-HNL). Fraction of exhaled nitric oxide (FeNO), blood eosinophil (B-Eos) count, C-reactive protein (CRP), airway responsiveness to methacholine (PD_20_), and asthma-related quality of life (mAQLQ) were also measured.

**Results:**

NAA had lower FeNO (*p* < 0.001) and B-Eos count (*p* < 0.001), but scored worse on mAQLQ (*p* = 0.045) compared with AA. NAA displayed higher levels of matrix metalloproteinase-1 (MMP-1) compared with both NAC (*p* = 0.011) and AA (*p* = 0.001), and lower PD_20_ compared with NAC (*p* < 0.001). In NAA, S-HNL correlated negatively with PD_20_ (rho = − 0.048, *p* < 0.05) and CRP correlated negatively with mAQLQ (rho = − 0.439, *p* < 0.05).

**Conclusion:**

In a subgroup of non-atopic young asthmatics with perceived cow’s milk hypersensitivity we observed poor asthma-related quality of life, airway hyperresponsiveness, and clinically relevant non-type 2 inflammation. MMP-1 was elevated in this group, which deserves further studies.

**Electronic supplementary material:**

The online version of this article (10.1186/s13601-019-0250-2) contains supplementary material, which is available to authorized users.

## Introduction

Asthma associated with elevated type 2 immune responses is termed type 2 asthma, and is often related to atopy, especially in young subjects [1]. It is well-known that elevated fraction of exhaled nitric oxide (FeNO) and blood eosinophil (B-Eos) count are clinically useful type 2 biomarkers in asthma, and their simultaneous elevation has shown independent and additive predictive value for asthma morbidity [2, 3]. We have recently reported the persistence of clinically relevant type 2 inflammatory signals in young asthmatics with IgE antibody concentrations below 0.35 kU_A_/L, and we have suggested a cut-off of 0.10 kU_A_/L for ruling out type 2 inflammation [4].

Less progress has been made in defining non-type 2 asthma and few biomarkers have been described for this endotype. This type of asthma primarily affects older subjects [5], although its presence has also been described among young subjects [6]. It is likely that non-type 2 asthma includes more than one endotype and proven treatment options are limited. For example, non-type 2 (non-eosinophilic) asthma is less responsive to corticosteroids [7] and implicates the activation of neutrophils [8].

We have recruited a large sample of young (10–35 years) patients with asthma, from both primary and secondary care: the MIDAS cohort [2, 9]. Within this cohort, we have identified a non-atopic subgroup with perceived food hypersensitivity that showed lower asthma-related quality of life and poorer asthma control compared with both atopic asthmatics and non-atopic asthmatics without reported food hypersensitivity [6]. These subjects were characterized by low FeNO, suggesting non-type 2 asthma.

True non-type 2 asthma in young subjects has scarcely been investigated. In this study, our aim was to explore a biomarker pattern that could help define inflammatory mechanisms in these subjects with non-atopic asthma and perceived food hypersensitivity.

## Methods

### Patients

The Minimally-Invasive Diagnostic procedures in allergy, Asthma, or food hypersensitivity Study (MIDAS) asthma cohort was recruited from primary and specialist care facilities in Uppsala, Sweden [9, 10]. This cohort comprises 411 subjects aged 10–35 years, and the present study was initially based on 405 subjects with complete datasets, including IgE measurements and asthma control scores. The inclusion criteria were physician-diagnosed asthma and daily treatment with an inhaled corticosteroid (ICS) and/or an oral leukotriene-receptor antagonist (LTRA) during at least three of the past twelve months. The cohort also included 118 age- and sex-matched non-asthmatic controls randomly chosen from the population registry.

### Grouping based on presence of asthma and IgE sensitization

**Fig. 1:**
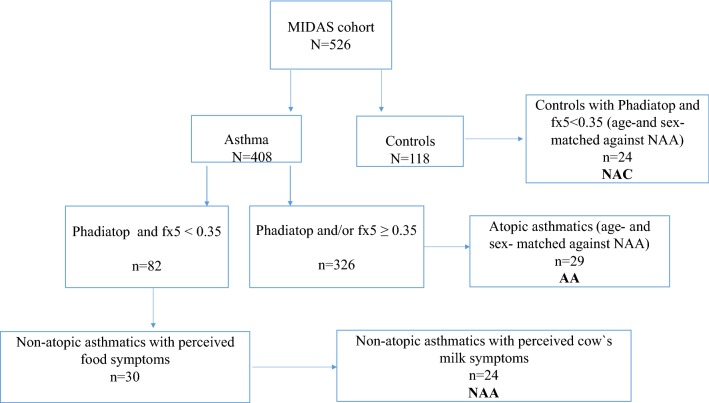
Description of the included study groups as regards IgE sensitization and perceived food hypersensitivity. IgE concentrations in kU_A_/L

Three subgroups were formed from this cohort with regard to history of asthma, food hypersensitivity, and atopy. The first group consisted of all asthmatics with perceived cow’s milk hypersensitivity, but without clear IgE sensitization to aeroallergens and food allergens (< 0.35 kU_A_/L), denoted non-atopic asthmatics (NAA, n = 24). NAA were extracted from a slightly larger group of non-atopic asthmatics with a history of perceived food hypersensitivity (see below). The second group consisted of control subjects without asthma and without clear IgE antibody sensitization (< 0.35 kU_A_/L) to aeroallergens and food allergens (non-atopic controls, NAC, n = 24). The third group consisted of atopic asthmatics (AA) with an IgE antibody concentration to aeroallergens ≥ 0.35 kU_A_/L (n = 29). The subjects in the three groups were age- and sex-matched (Fig. 1).

### Perceived food hypersensitivity symptoms

Each subject was asked to report any history of hypersensitivity reactions to food (egg white, cod fish, milk, soybean, wheat, or peanut). Perceived symptoms were grouped based on the organ affected: the lower airways (asthma), the upper airways (rhinitis, conjunctivitis), the oral cavity (oral allergy syndrome), the skin (atopic dermatitis, urticaria, and angioedema), the gastrointestinal tract (GI) (nausea, vomiting, abdominal pain, diarrhoea), and anaphylaxis (including self-reported). Out of the 30 non-atopic subjects with asthma with perceived food hypersensitivity, 24 reported cow’s milk hypersensitivity, three subjects wheat hypersensitivity, two subjects peanut hypersensitivity, and one subject had perceived cod fish hypersensitivity. We focused on the 24 subjects (80%) who reported cow’s milk hypersensitivity (NAA), based on the long-standing clinical recognition of this particular phenotype by one of the co-authors (LN), and for the sake of homogeneity.

### Blood measurements

The presence of IgE antibodies was examined using a mix of nine aeroallergens (birch, timothy grass, mugwort, *Dermatophagoides pteronyssinus*, *Dermatophagoides farinae*, *Cladosporium herbarum*, cat, dog, and horse; Phadiatop) and a mix of six food allergens (egg white, cod fish, milk, soybean, wheat, and peanut; fx5) [11]. Serum IgE antibodies, total IgE, IgG/IgA antibodies and eosinophilic cationic protein (S-ECP) were measured in the ImmunoCAP system (ImmunoDiagnostics, Thermo Fisher Scientific, Uppsala, Sweden). Subjects were considered to be atopic if they had IgE antibody concentrations ≥ 0.35 kU_A_/L.

Serum human neutrophil lipocalin (S-HNL) concentrations were measured in an ELISA (Diagnostics Development, Uppsala, Sweden). Plasma was analysed using a panel of 92 inflammation-related proteins (Proseek Multiplex Inflammation; Olink Bioscience, Uppsala, Sweden) [12]. The inflammation panel was based on a multiplex proximity extension immunoassay (PEA), enabling analysis of 92 proteins simultaneously. The data were given in the form of relative quantification and expressed as normalized protein expression (NPX). Further, plasma C-reactive protein (P-CRP) was analysed using an immunoturbidimetric method (CRP Vario, Abbott, Illinois, USA), and blood cells were counted using an automated cell counter (Cell-Dyn Sapphire, Abbott, Illinois, USA); both analyses were performed at Uppsala University Hospital.

### Asthma symptoms, attacks and medication

The degree of asthma control was assessed using the Asthma Control Test (ACT) [13]. The total ACT score is between 5 and 25, with a lower score indicating poorer asthma control. A score ≥ 20 indicates well-controlled asthma. The mini asthma quality of life questionnaire (mAQLQ) consists of 15 questions. The score ranges from 1 to 7, with a lower score indicating a poorer quality of life [14]. Use of ICS and LTRA was recorded in the interviews. Asthma symptoms during the last 12 months were also recorded [15]. Subjects were divided into groups based on whether or not they had had a recent asthma attack (last three months, self-reported).

### Exhaled nitric oxide

The fraction of exhaled nitric oxide (FeNO) was measured in accordance with the American Thoracic Society (ATS)/European Respiratory Society recommendations [16] using a chemiluminescence analyser at an exhalation flow rate of 50 mL/s (NIOX Flex; Aerocrine AB, Solna, Sweden). The mean value from three exhalations (or two, if the first two NO measurements were within 10% of each other) was used for statistical analysis.

### Lung function

Forced expiratory volume in one second (FEV_1_) was measured using a Masterscope spirometer (Viasys Healthcare GmbH, Höchberg, Germany). Recommendations from the ATS were followed [17]. The subjects were subdivided into groups with reduced or normal lung function (FEV_1_ < 80% or ≥ 80%). The percent of predicted values for FEV_1_ were calculated on the basis of Hedenström reference values for those ≥ 18 years of age [18, 19] and Solymar reference values for subjects < 18 years of age [20]. A bronchial provocation test was performed with Aerosol Provocation System (Viasys Healthcare GmbH, Höchberg, Germany) using a single concentration of methacholine and increasing doses up to a maximal cumulative dose of 3.63 mg methacholine. Airway responsiveness was defined as normal if the cumulative dose causing a reduction in FEV_1_ of 20% (PD_20_) was > 1.0 mg, borderline-to-mild at 0.3–1.0 mg, and moderate-to-severe at < 0.3 mg, in accordance with Schulze et al. [21].

### Statistics

All statistical analyses were performed using STATA/IC 13.1 (StataCorp LP, College Station, Texas, USA). If continuous variables had a distribution skewed to the right, a geometric mean with a 95% confidence interval was used for descriptive statistics and Mann–Whitney U test (for continuous variables) and Fisher’s exact test (for categorical variables) were used for group comparisons. Seven subjects with fx5 between 0.10 and 0.34 kU_A_/L and three subjects with Phadiatop between 0.10 and 0.34 kU_A_/L were found in the NAA group, while the remaining 14 had both Phadiatop and fx5 below 0.10 kU_A_/L. We generated a subgroup based on detectable IgE levels between 0.10 and 0.34 kU_A_/L and compared it to the rest of the NAA group.

Multivariate ANOVA with concomitant adjustment for confounders: age, gender, body mass index (BMI), and smoking was used in group comparisons for candidate proteins, and the robustness of the test was evaluated using bootstrapping. The *p* values were adjusted for multiple testing using the Benjamin-Hochberg procedure. This procedure is based on controlling the false discovery rate and limiting that to a predefined value (0.05) [22]. We performed exploratory correlation analyses between the different biomarkers, including the significant inflammation-related proteins, and clinical outcomes (mAQLQ, ACT, PD_20_, recent asthma attacks) in each group, using the Spearman’s test. A *p* value < 0.05 was considered statistically significant and *p* < 0.10 indicated a trend.

## Results

### Subject characteristics

NAA had increased airway responsiveness to methacholine, reduced FEV_1_/FVC ratio, and were shorter than NAC (Table 1). Further, NAA had lower levels of type 2 biomarkers, but scored worse on mAQLQ compared with AA. No differences in blood total leukocyte, monocyte, lymphocyte, or basophil counts were noted between the groups (data not shown). Asthma medication use, lung function, airway responsiveness, and asthma symptoms were similar in NAA and AA. The clinical and inflammatory outcomes were not significantly different between the subgroup of NAA with detectable IgE levels (0.10–0.34 kU_A_/L) and the rest of the NAA group (data not shown). Cow’s milk-induced symptoms from the GI tract were present among all NAA, but only in 10% (n = 3) of the AA group (Additional file 1: Table E1). NAA did not report any anaphylaxis or symptoms from the upper airways and oral cavity, but had higher prevalence of milk-induced other symptoms compared with AA. NAA had lower total IgG antibody concentrations to α-lactalbumin and β-lactoglobulin, and lower IgG4 to α-lactalbumin than AA (Table 2). Furthermore, NAA had lower IgA antibody concentration and a trend for lower IgG to casein than NAC. In the larger group with non-atopic asthma (n = 82, see Fig. 1), subjects with any perceived food symptoms (n = 30) scored worse on ACT and mAQLQ when compared to subjects without food symptoms, whereas no other differences in the studied inflammatory or clinical outcomes were observed between these two groups (Additional file 1: Table E2).

**Table 1:** Characterization of the three groups included in the study. Comparison of the non-atopic asthmatics (NAA) to non-atopic controls (NAC) and atopic asthmatics (AA)

	NAA (n = 24)	NAC (n = 24)	AA (n = 29)	*p* value NAA-NAC	*p* value NAA-AA
Females (%)	60	54.1	48.2	0.687	0.398
Age (years)	18.5 ± 5.90	19.5 ± 5.98	18.4 ± 5.80	0.526	0.946
Height (cm)	163 ± 13.6	172 ± 11.5	166 ± 14.3	0.021	0.553
Weight (kg)	59.3 ± 15.9	65.9 ± 16.2	61.6 ± 19.3	0.161	0.646
FeNO (ppb)	9.78 (8.15, 11.7)	10.5 (9.01, 12.3)	29.2 (19.4, 44.2)	0.534	< .001
B-Eos	0.118 (0.088, 0.159)	0.086 (0.067, 0.110)	0.357 (0.257, 0.498)	0.097	< .001
B-Neu	3.23 (2.68, 3.90)	3.13 (2.65, 3.70)	3.26 (2.85, 3.72)	0.796	0.941
Phadiatop	0.053 (0.041, 0.069)	0.046 (0.037, 0.057)	22.8 (12.5, 41.6)	0.349	< .001
fx5	0.054 (0.038, 0.073)	0.053 (0.038, 0.073)	2.77 (0.963, 7.98)	0.627	< .001
Total IgE	22.8 (14.1, 36.6)	15.6 (8.81, 27.7)	458 (334, 628)	0.300	< .001
S-ECP	9.76 (7.62, 12.5)	8.33 (6.02, 11.5)	20.0 (14.9, 26.9)	0.423	< .001
P-CRP	0.510 (0.267,0.973)	0.522 (0.288, 0.945)	0.551 (0.327, 0.928)	0.956	0.846
S-HNL	75.2 (63.0, 89.7)	73.4 (62.7, 86.0)	72.1 (63.6, 81.8)	0.835	0.684
Smoking (%)	0	8.33	6.89	0.146	0.187
ICS (µg)	438 (312, 615)	–	395 (307, 509)	–	0.876
LTRA (%)	24.0	–	20.7	–	0.999
ACT	18.8 ± 4.51	–	20.0 ± 2.89	–	0.140
mAQLQ	5.17 ± 1.20	–	5.74 ± 0.821	–	0.045
FEV_1_ (%)	92.7 ± 12.5	98.0 ± 13.5	91.1 ± 16.3	0.156	0.701
FEV_1_ < 80 (%)	16.0	4.17	24.1	0.178	0.468
FEV_1_/FVC	82.6 (79.5, 85.8)	87.7 (85.4, 90.1)	80.5 (76.8, 84.2)	0.011	0.402
PD_20_ (mg)	0.677 (0.297, 1.54)	4.56 (3.25, 6.41)	0.393 (0.130, 1.19)	< .001	0.422
Recent asthma attacks	56.0	–	48.2	–	0.320

Mean ± SD, Geometric mean (95% CI). IgE concentrations in kU_A_/L, white blood cells in × 10^9^/L, P-CRP in mg/L, and S-ECP and S-HNL in µg/L

**Table 2:** IgG and IgA antibody concentrations (mg/L) in the included study groups. Comparisons of non-atopic asthmatics (NAA) with non-atopic controls (NAC) and atopic asthmatics (AA)

	NAA (n = 24)	NAC (n = 24)	AA (n = 29)	*p* value NAA-NAC	*p* value NAA-AA
IgG α-LA	1.11 (0.724, 1.72)	1.46 (0.942, 2.27)	3.87 (2.26, 6.63)	0.379	0.001
IgG β-LG	2.43 (1.42, 4.14)	2.50 (1.38, 4.52)	7.31 (4.24, 12.6)	0.975	0.017
IgG casein	7.07 (5.07, 9.86)	10.3 (7.24, 14.7)	9.97 (7.43, 13.3)	0.085	0.078
IgG4 α-LA	0.200 (0.086, 0.461)	0.123 (0.047, 0.318)	0.975 (0.405, 2.34)	0.968	0.002
IgG4 β-LG	0.805 (0.330, 1.96)	0.499 (0.186, 1.33)	2.77 (1.47, 5.22)	0.529	0.052
IgG4 casein	1.20 (0.698, 2.08)	1.18 (0.669, 2.09)	1.55 (0.974, 2.48)	0.826	0.497
IgA α-LA	0.520 (0.479, 0.565)	0.500 (0.500, 0.500)	0.522 (0.477, 0.570)	0.327	0.936
IgA β-LG	0.520 (0.479, 0.564)	0.540 (0.483, 0.603)	0.566 (0.500, 0.641)	0.531	0.234
IgA casein	0.819 (0.581, 1.15)	1.37 (0.978, 1.94)	0.921 (0.681, 1.24)	0.014	0.471

α-LA = α-lactalbumin, β-LG = β-lactoglobulin. Geometric mean (95% CI)

### Exploratory proteomic analysis and correlation between inflammatory markers

**Fig. 2:**
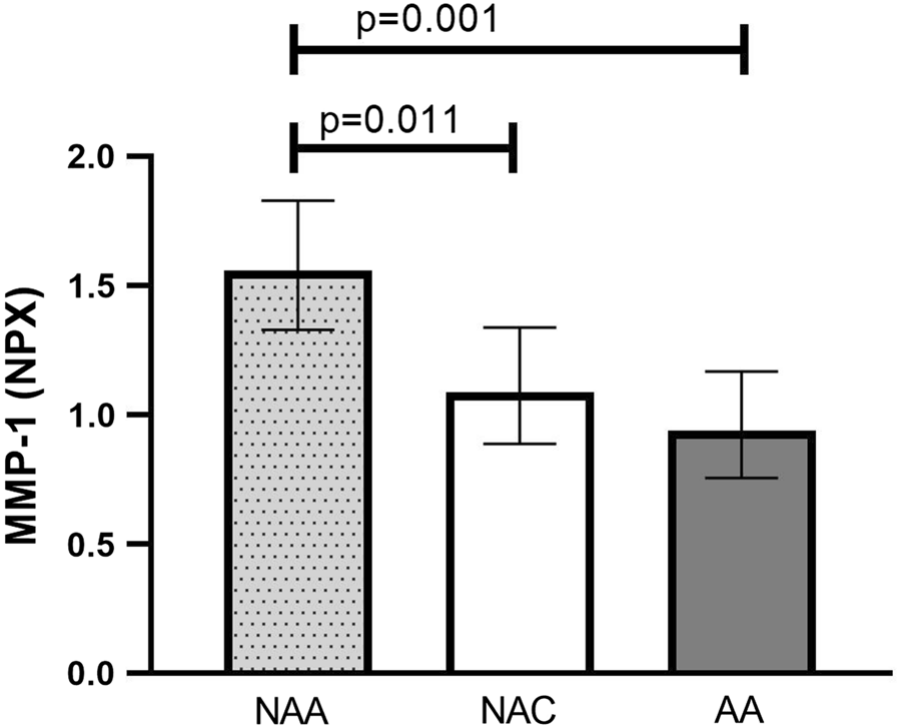
Plasma levels of MMP-1 in the included study groups. Geometric mean (95% CI)

For 79 out of 92 proteins (86%) in the Proseek panel, at least 50% of the observations were above the detection limit, and these were included in further analyses (see Additional file 1: Table E3). Three proteins showed significant group differences in an ANOVA adjusted for confounders (crude *p* value < 0.05): MMP-1, FGF5, and IL-10. After correction for multiple testing, MMP-1 was the only protein that maintained a trend (*p* = 0.10). Based on this, we performed correlation analyses between MMP-1 and all other inflammation markers in the NAA group: MMP-1 correlated significantly with FeNO, CRP, IL-8, IL-20, and CXCL9 (Table 3). When repeating the correlation analysis for these proteins in AA and NAC, MMP-1 was found to correlate with CRP and IL-20 in AA and with CRP in NAC. IL-6 correlated with CRP in NAA (rho = 0.422, *p* = 0.035), but IL-6 did not correlate with MMP-1 in this group (rho = 0.055, *p* = 0.792). Furthermore, in NAA, CXCL9 correlated significantly with FeNO (Additional file 1: Table E4). MMP-1 was higher in NAA compared with both NAC and AA (Fig. 2), and IL-8 was found to be higher in NAA than in NAC, whereas CXCL9 was significantly lower in NAA than AA (Table 4).

**Table 3:** Correlations (rho) between MMP-1 and different inflammatory markers in the included study groups

*MMP*-*1*	FeNO	CRP	IL-8	IL-20	CXCL9
NAA	0.509*	0.478*	0.553*	0.407*	0.457*
NAC	− 0.337	0.541*	0.077	− 0.066	0.095
AA	− 0.035	0.465*	0.314^(^*^)^	0.387*	0.045

*p < 0.05, ^(^*^)^*p* < 0.10

**Table 4:** Group comparisons for the proteins significantly correlating with MMP-1 (concentrations in NPX)

	NAA (n = 24)	NAC (n = 24)	AA (n = 29)	*p* value NAA-NAC	*p* value NAA-AA
IL-8	5.13 (4.98, 5.28)	4.84 (4.65, 5.04)	5.00 (4.87, 5.13)	0.032	0.160
IL-20	0.255 (0.180, 0.363)	0.262 (0.182, 0.378)	0.259 (0.185, 0.363)	0.706	0.739
CXCL9	5.64 (5.41, 5.87)	5.72 (5.51, 5.94)	6.03 (5.78, 6.30)	0.660	0.039

Geometric mean (95% CI)

### Correlations between potential biomarkers and clinical outcomes

In NAA, S-HNL correlated negatively with PD_20_ (Table 5). Furthermore, significant negative correlations were noted between CRP, and ACT and mAQLQ scores, respectively. There were also trends for significant negative correlations between the ACT score, and MMP-1 and IL-8, respectively, as well as between mAQLQ and B-Neu. No associations between type 2 biomarkers and clinical outcomes were found in the NAA group. In AA, the type 2 biomarkers FeNO, B-Eos, and S-ECP all correlated negatively with PD_20_, and total IgE correlated negatively with FEV_1_ (Table 6). MMP-1 showed a weak but significant negative correlation with ACT score, whereas CRP correlated positively with PD_20_, and B-Neu showed a similar trend in this group.

**Table 5:** Correlations (rho) between clinical outcomes and inflammatory markers in NAA

*NAA*	ACT	mAQLQ	PD_20_	FEV_1_	FEV_1_/FVC
P-CRP	− 0.402*	− 0.439*	− 0.299	− 0.294	− 0.043
S-HNL	0.144	− 0.030	− 0.048*	0.243	0.238
B-Neu	− 0.323	− 0.387^(^*^)^	− 0.126	0.042	0.138
B-Eos	0.268	0.003	− 0.333	0.192	− 0.095
S-ECP	0.030*	0.175	− 0.332	0.294	0.078
Total IgE	0.008	0.216	0.075	0.420	0.189
MMP-1	− 0.359^(^*^)^	− 0.320	− 0.030	− 0.020	0.255
IL-8	− 0.358^(^*^)^	− 0.266	0.079	− 0.122	− 0.152
IL-20	− 0.131	− 0.089	0.336	− 0.047	0.525
CXCL9	0.059	− 0.054	− 0.183	0.009	− 0.151
FeNO	− 0.081	− 0.003	− 0.152	0.072	− 0.108

*p < 0.05, ** *p* < 0.01, ^(^*^)^*p* < 0.10

**Table 6:** Correlations (rho) between clinical outcomes and inflammatory biomarkers in AA

*AA*	ACT	mAQLQ	PD_20_	FEV_1_	FEV_1_/FVC
P-CRP	− 0.089	− 0.018	0.421*	0.142	− 0.038
S-HNL	− 0.075	− 0.142	0.213	0.226	− 0.134
B-Neu	− 0.051	0.100	0.365^(^*^)^	0.163	0.096
B-Eos	− 0.217	− 0.103	− 0.788**	− 0.186	− 0.213
S-ECP	− 0.083	− 0.219	− 0.662**	− 0.163	− 0.157
Total IgE	− 0.046	0.357	− 0.134	− 0.651*	0.236
MMP-1	− 0.038*	− 0.225	0.184	0.162	− 0.100
IL-8	− 0.207	0.150	− 0.064	− 0.068	0.015
IL-20	− 0.116	− 0.233	0.057	0.158	0.139
CXCL9	0.128	0.435	− 0.343	0.006	− 0.165
FeNO	− 0.049	− 0.011	− 0.725**	− 0.052	− 0.291

*p < 0.05, **p < 0.01, ^(^*^)^*p* < 0.10

## Discussion

The main finding of this study was that in non-atopic asthmatics with perceived cow’s milk hypersensitivity, despite being free from type 2 inflammation, we observed airway hyper-responsiveness, reduced FEV_1_/FVC ratio, and lower asthma-related quality of life than in those with atopic asthma. Furthermore, in this group, airway responsiveness, asthma control, and asthma-related quality of life all associated with signs of non-type 2 inflammation, such as blood neutrophil count, CRP, HNL, IL-8, and MMP-1. MMP-1 was the biomarker best distinguishing this group of non-atopic asthmatics from both controls and atopic asthmatics.

In non-atopic asthmatics reporting hypersensitivity reactions to cow’s milk, correlations between type 2 biomarkers and clinical outcomes were absent. Instead, correlations were observed between HNL and methacholine responsiveness, and between CRP and both mAQLQ and ACT; such correlations were absent in the atopic group, indicating an immunological background distinct from the type 2 spectrum. HNL and CRP have mainly been used as diagnostic tools for distinguishing bacterial from viral infections [23, 24]. However, a few studies have associated HNL and CRP to airway inflammation and to the prediction of asthma status [25–27], but these markers seem to be resistant to corticosteroid treatment [28]. Several studies have emphasized the role of neutrophilic inflammation in corticosteroid-resistant asthma involving IL-8 as a potent mediator [29–31]. Since corticosteroids may even promote neutrophil survival [32], attempts targeting neutrophilic inflammation have been made, but with only minor clinical effects, and validated biomarkers are lacking at the moment [1, 33]. Our results revealed airway hyper-responsiveness and systemic inflammation in non-atopic subjects with ongoing asthma medication, indicating the need for new therapeutic strategies.

In contrast, the atopic asthmatics showed higher levels of all type 2 biomarkers (FeNO, B-Eos, S-ECP, and IgE) than non-atopic asthmatics. Furthermore, the degree of type 2 inflammation correlated with clinical outcomes. For example, FeNO, B-Eos, and S-ECP correlated with methacholine responsiveness. Interestingly, MMP-1 was found to correlate negatively with ACT score, but not with type 2 biomarkers, in this group of atopic asthmatics. Furthermore, CRP and blood neutrophil count associated positively with methacholine reactivity, indicating a down-regulation of innate mechanisms by type 2 inflammation.

Studies have suggested that some biomarkers are implicated in both type 2 and non-type 2 inflammatory pathways, making the distinction more complicated. For example, matrix metalloproteinases (MMPs) are endopeptidases that are involved in the pathophysiology of airway inflammation in allergic asthma, but they may also be involved in monocyte recruitment to inflammatory sites [34, 35]. Furthermore, MMPs has been implicated in the pathophysiology of intestinal inflammation by promoting neutrophil recruitment in the intestinal wall and activating T cells, leading to gastrointestinal damage [36]. Interestingly, it was recently shown that plasma MMP-1 was reduced after the introduction of exclusive enteral nutrition in patients with juvenile idiopathic arthritis, another disease where the involvement of the GI tract has been suggested [37]. A novel finding of the present study was that MMP-1 was the biomarker most significantly elevated in non-atopic subjects as compared with in either atopic asthmatics or non-atopic controls. According to other studies, MMP-1 expression is believed to be stimulated by type 2 cytokines and thereby contributes to airway hyper-responsiveness in allergen-induced asthma [38, 39]. Moreover, the possible activation of MMP-1 by mast cell tryptase could result in airway hyper-responsiveness and airway narrowing [40]. Interestingly, in the non-atopic asthma group, FeNO, MMP-1, and CXCL9 all inter-correlated, whereas this was not seen in atopic asthmatics. CXCL9, a chemoattractant for T lymphocytes, involving elements of mixed type 2 and non-type 2 bronchial inflammation in asthma, is upregulated by IFN-γ, a Th1 cytokine [41–43]. In addition, it has previously been suggested that basal NO formation in the respiratory epithelium is dependent on homeostatic IFN-γ [44]. However, an IFN-γ-mediated basal regulation of airway NO formation may not be easily detected in atopic asthmatics, where IL-4/IL-13-driven NO formation will dominate [44]. Thus, we suggest that the association between FeNO and plasma CXCL9, and possibly MMP-1, depends on the expression of IFN-γ. No significant correlations between these biomarkers and IFN-γ were noted in this study, but this could be due to many observations being below the detection limit for IFN-γ (close to 50%). Furthermore, we could show that MMP-1 correlated with CRP, but not IL-6, in non-atopic asthmatics, whereas IL-6 and CRP correlated with each other as expected [45]. This may indicate that the association between MMP-1 and CRP relies on IL-1β and/or tumour necrosis factor (TNF), both of which are also involved in CRP induction. However, this could not be confirmed in the present study since IL-1β was not part of the Proseek panel, and TNF was below the detection limit in more than 50% of the observations. However, as a support to this hypothesis, IL-20, which is known to be induced by IL-1β and TNF [46], correlated with MMP-1 in non-atopic asthmatics in the present study. IL-20 has also been suggested to be involved in airway remodelling in asthma [47]. The fact that MMP-1 correlated with quite diverse inflammation markers including FeNO, IL-8, CRP etc., implicates multiple cellular origin of this proteinase.

An interesting feature in the non-atopic group with perceived milk hypersensitivity was the poorer asthma-related quality of life as compared with the atopic asthma group. We have previously published this in non-atopic subjects reporting any food hypersensitivity [6], and could now confirm this finding in the more homogenous subgroup specifically reporting milk hypersensitivity. The present results further strengthen the earlier observations, since we managed to objectively confirm the presence of airway disease including airway hyper-responsiveness and reduced FEV_1_/FVC ratio. We have recently reported data showing weak type 2 inflammatory signals in young asthmatics with IgE antibody concentrations below 0.35 kU_A_/L, suggesting a cut-off of 0.10 kU_A_/L for ruling out the presence of such inflammation [4]. In the present study, 58% of the subjects that reported hypersensitivity to cow’s milk had undetectable IgE antibody concentrations (< 0.10 kU_A_/L). When the subgroup with detectable IgE (0.10–0.34 kU_A_/L) was compared with the subjects with undetectable IgE, no differences in clinical or inflammatory indices were noted. We assume that the distribution of IgE levels in this particular group of asthmatics is similar to that in the normal population, but that the low IgE antibody concentrations do not reflect mechanisms driving the asthma disease, just as they do not in healthy controls.

Non-IgE-mediated cow’s milk hypersensitivity characterized the studied group. We also examined IgG and IgA antibodies against different cow’s milk proteins, but no elevated levels were found in the non-atopic asthmatics. Instead, lower antibody levels against casein were seen than in controls, which could be the consequence of lower consumption of milk due to the perceived symptoms.

A strength of the present study was the recruitment of young asthmatics from both primary and secondary care units. Three approximately equal-sized subgroups that were age- and sex-matched were created and both non-atopic healthy controls and atopic asthmatics were used for comparisons. However, this could also be considered a limitation due to the relatively small number of subjects in each group, even though the subjects that were hypersensitive to cow’s milk were taken from a large sample of asthmatics (n = 408). Another limitation of this study may be that the cow’s milk hypersensitivity was self-reported and not confirmed in provocation tests. One should be aware of the similarity of the symptoms between non-IgE-mediated cow’s milk hypersensitivity and other gastrointestinal disturbances, such as lactose intolerance, gastrointestinal infections, or irritable bowel syndrome. However, very few subjects reported gastrointestinal disorders other than perceived food hypersensitivity in the interviews.

In conclusion, we found that non-atopic subjects with cow’s milk hypersensitivity constituted almost 6% of a representative sample of young asthmatics. These asthmatics were characterized by airway hyper-responsiveness, reduced lung function, and poor asthma-related quality of life. Biomarkers, including those not extensively investigated previously, showed clear signs of clinically relevant non-type 2 inflammation. Future studies may help us understand how these biomarkers might direct towards more appropriate treatment of young subjects with non-atopic asthma.

## Additional file

**Additional file 1. Table E1** Prevalence (%) of perceived food and cow´s milk hypersensitivity symptoms in the three groups included in the study. Fischer´s exact test was performed for comparison of the proportion of perceived cow´s milk symptoms in the NAA and AA groups. **Table E2** Comparison of non-atopic asthmatics with any perceived food hypersensitivity with non-atopic asthmatics without food symptoms. **Table E3** List of the inflammation-related proteins included in the study (Proseek Inflammation,Olink). p values for the adjusted ANOVA comparing the three groups before and after correction for multiple testing according to the Benjamin-Hochberg* procedure. See methods for more information. **Table E4** Correlations (rho) between inflammatory markers in NAA. * p < 0.05, (*) p < 0.10.

## Abbreviations

AA: atopic asthmatics
ACT: Asthma Control Test
ATS: American Thoracic Society
B-Eos: blood eosinophil
B-Neu: blood neutrophil
CXCL: C-X-C motif chemokine ligand
FeNO: fraction of exhaled nitric oxide
FEV_1_: forced expiratory volume in 1 s
FGF: fibroblast growth factor
ICS: inhaled corticosteroid
IFN-γ: interferon gamma
IgE: immunoglobulin E
IL: interleukin
α-LA: α-lactalbumin
β-LG: β-lactoglobulin
LTRA: leukotriene-receptor antagonist
mAQLQ: mini Asthma Quality of Life Questionnaire
MMP: matrix metalloproteinase
NAA: non-atopic asthmatics
NAC: non-atopic controls
NPX: normalized protein expression
PD_20_: provocative dose causing a fall in FEV_1_ by 20%
PEA: Proximity Extension Assay
P-CRP: plasma C-reactive protein
ppb: parts per billion
S-ECP: serum eosinophilic cationic protein
S-HNL: serum human neutrophil lipocalin
Th: T helper
