# Supplementary material for: Characterization of a subgroup of non-type 2 asthma with cow’s milk hypersensitivity in young subjects
Source: Clin Transl Allergy. 2019 Feb 22;9:12. doi: 10.1186/s13601-019-0250-2 (PMC6385459; doi:10.1186/s13601-019-0250-2)
Supplement: Supplementary file 1 — Additional file 1. Table E1 Prevalence (%) of perceived food and cow´s milk hypersensitivity symptoms in the three groups included in the study. Fischer´s exact test was performed for comparison of the proportion of perceived cow´s milk symptoms in the NAA and AA groups. Table E2 Comparison of non-atopic asthmatics with any perceived food hypersensitivity with non-atopic asthmatics without food symptoms. Table E3 List of the inflammation-related proteins included in the study (Proseek Inflammation,Olink). p values for the adjusted ANOVA comparing the three groups before and after correction for multiple testing according to the Benjamin-Hochberg* procedure. See methods for more information. Table E4 Correlations (rho) between inflammatory markers in NAA. * p < 0.05, (*) p < 0.10. [file 13601_2019_250_MOESM1_ESM.docx]

Table E1. Prevalence (%) of perceived food and cow’s milk hypersensitivity symptoms in the three groups included in the study. Fischer's exact test was performed for comparison of the proportion of perceived cow's milk symptoms in the NAA and AA groups.

| % | NAA (n = 24) | | AA (n = 29) | | p NAA-AA (milk symptoms) | NAC (n = 24) | |
| --- | --- | --- | --- | --- | --- | --- | --- |
|  | Milk | Any food | Milk | Any food |  | Milk | Any food |
| Lower airways | 8 | 13 | 4 | 84 | 0.372 | 0 | 0 |
| Upper airways | 0 | 0 | 7 | 29 | - | 0 | 0 |
| GI | 100 | 100 | 10 | 70 | 0.001 | 0 | 0 |
| Skin | 4 | 10 | 7 | 20 | 0.537 | 0 | 0 |
| Oral cavity | 0 | 29 | 3 | 88 | - | 0 | 4 |
| Anaphylaxis | 0 | 0 | 3 | 28 | - | 0 | 0 |
| Other | 13 | 13 | 3 | 12 | 0.016 | 0 | 17 |

Table E2. Comparison of non-atopic asthmatics with any perceived food hypersensitivity with non-atopic asthmatics without food symptoms.

|  | NAA with food symptoms (n=30) | NAA without food symptoms (n=52) | p |
| --- | --- | --- | --- |
| Females (%) | 65 | 62 | 0.859 |
| Age (years) | 19.2 ± 1.54 | 21.6 ± 7.27 | 0.058 |
| Height (cm) | 164 ± 11.3 | 166 ± 11.2 | 0.386 |
| Weight (kg) | 62.3 ± 15.1 | 64.4 ± 14.4 | 0.157 |
| FeNO (ppb) | 9.80 (8.31, 11.6) | 10.7 (8.79,13.1) | 0.569 |
| B-Eos | 0.120 (0.088, 0.150) | 0.125 (0.100,0.155) | 0.776 |
| B-Neu | 3.21 (2.79, 3.69) | 3.12 (2.82,3.44) | 0.467 |
| Phadiatop | 0.062 (0.051, 0.076) | 0.073 (0.059,0.090) | 0.091 |
| fx5 | 0.055 (0.045, 0.066) | 0.060 (0.051,0.070) | 0.461 |
| Total IgE | 25.1 (16.3, 37.7) | 21.1 (14.6,30.4) | 0.809 |
| S-ECP | 9.23 (7.53, 11.3) | 9.84 (8.47,11.4) | 0.952 |
| P-CRP | 0.597 (0.366,0.973) | 0.658 (0.481,0.900) | 0.417 |
| S-HNL | 76.1 (63.9, 80.8) | 77.8 (71.4,84.8) | 0.691 |
| Smoking (%) | 3.21 | 1.7 | 0.509 |
| ICS (µg) | 414 (301, 569) | 389 (331,458) | 0.927 |
| LTRA (%) | 24.4 | 32.7 | 0.425 |
| ACT | 18.7 ± 0.812 | 20.4 ± 0.511 | 0.007 |
| mAQLQ | 5.18 ± 0.210 | 5.80 ± 1.01 | 0.001 |
| FEV_1_ (%) | 94.2 ± 12.8 | 90.15 ±15.2 | 0.466 |
| FEV_1_ < 80 (%) | 14.6 | 18.9 | 0.751 |
| FEV_1_/FVC | 83.6 ± 7.57 | 80.3 ± 9.42 | 0.226 |
| PD_20_ (mg) | 0.881 (0.233, 1.83) | 0.909 (0.549,1.50) | 0.387 |
| Recent asthma attacks (%) | 58.4 | 41.4 | 0.220 |

Mean ± SD, Geometric mean (95% CI). IgE concentrations in kU_A_/L, white blood cells in x10^9^/L, P-CRP in mg/L, and S-ECP and S-HNL in µg/L.

Table E3. List of the inflammation-related proteins included in the study (Proseek Inflammation, Olink). p values for the adjusted ANOVA comparing the three groups before and after correction for multiple testing according to the Benjamin-Hochberg^*^ procedure. See Methods for more information.

| Protein | Crude p value | | *BH-corrected p value |
| --- | --- | --- | --- |
| MMP1 | 0.00 | 0.10 | |
| FGF5 | 0.03 | 0.65 | |
| IL10 | 0.04 | 0.65 | |
| MIP1alpha | 0.05 | 0.65 | |
| CXCL9 | 0.05 | 0.65 | |
| FGF21 | 0.06 | 0.65 | |
| IL8 | 0.07 | 0.65 | |
| CCL4 | 0.08 | 0.65 | |
| MCP3 | 0.09 | 0.65 | |
| CD5 | 0.09 | 0.65 | |
| MMP10 | 0.11 | 0.65 | |
| MCP4 | 0.11 | 0.65 | |
| Flt3L | 0.13 | 0.65 | |
| IL18 | 0.14 | 0.65 | |
| TNFSF14 | 0.15 | 0.65 | |
| TGFA | 0.15 | 0.65 | |
| ENRAGE | 0.16 | 0.65 | |
| CXCL1 | 0.16 | 0.65 | |
| TNFB | 0.16 | 0.65 | |
| NRTN | 0.17 | 0.65 | |
| CXCL6 | 0.17 | 0.65 | |
| OSM | 0.18 | 0.65 | |
| CCL20 | 0.19 | 0.65 | |
| IL12B | 0.21 | 0.68 | |
| IL15RA | 0.22 | 0.68 | |
| CD40 | 0.22 | 0.68 | |
| MCP2 | 0.27 | 0.74 | |
| NT3 | 0.28 | 0.74 | |
| TWEAK | 0.30 | 0.74 | |
| IL17C | 0.32 | 0.74 | |
| IL7 | 0.33 | 0.74 | |
| CD244 | 0.34 | 0.74 | |
| CXCL10 | 0.34 | 0.74 | |
| CDCP1 | 0.35 | 0.74 | |
| CCL28 | 0.35 | 0.74 | |
| AXIN1 | 0.35 | 0.74 | |
| IL6 | 0.36 | 0.74 | |
| CXCL5 | 0.36 | 0.74 | |
| ADA | 0.40 | 0.81 | |
| TNFRSF9 | 0.43 | 0.81 | |
| IL10RA | 0.43 | 0.81 | |
| ST1A1 | 0.43 | 0.81 | |
| IL17A | 0.44 | 0.81 | |
| CCL19 | 0.47 | 0.85 | |
| CD6 | 0.51 | 0.89 | |
| SLAMF1 | 0.52 | 0.89 | |
| IFNgamma | 0.56 | 0.91 | |
| FGF23 | 0.57 | 0.91 | |
| IL10RB | 0.58 | 0.91 | |
| TRANCE | 0.61 | 0.91 | |
| LIFR | 0.61 | 0.91 | |
| CST5 | 0.62 | 0.91 | |
| OPG | 0.63 | 0.91 | |
| CX3CL1 | 0.63 | 0.91 | |
| SIRT2 | 0.64 | 0.91 | |
| 4EBP1 | 0.64 | 0.91 | |
| CASP8 | 0.67 | 0.93 | |
| IL13 | 0.69 | 0.93 | |
| CSF1 | 0.70 | 0.93 | |
| IL5 | 0.71 | 0.93 | |
| FGF19 | 0.72 | 0.93 | |
| hGDNF | 0.76 | 0.95 | |
| TRAIL | 0.77 | 0.95 | |
| CCL11 | 0.79 | 0.95 | |
| BDNF | 0.80 | 0.95 | |
| STAMPB | 0.81 | 0.95 | |
| MCP1 | 0.83 | 0.95 | |
| uPA | 0.83 | 0.95 | |
| CXCL11 | 0.84 | 0.95 | |
| HGF | 0.85 | 0.95 | |
| LAPTGFbeta1 | 0.86 | 0.95 | |
| CCL23 | 0.90 | 0.99 | |
| IL20 | 0.93 | 1.00 | |
| VEGFA | 0.93 | 1.00 | |
| DNER | 0.96 | 1.00 | |
| CCL25 | 0.98 | 1.00 | |
| SCF | 0.98 | 1.00 | |
| IL18R1 | 0.99 | 1.00 | |
| BetaNGF | 1.00 | 1.00 | |

Table E4. Correlations (rho) between inflammatory markers in NAA. ^*^ p < 0.05, ^(*)^ p < 0.10.

| *NAA* | CRP | FeNO | IL8 | IL20 | CXCL9 |
| --- | --- | --- | --- | --- | --- |
| CRP | - | 0.338^(*)^ | 0.183 | 0.359^(*)^ | 0.313 |
| FeNO | 0.338^(*)^ | - | 0.192 | 0.129 | 0.410^*^ |
| IL-8 | 0.183 | 0.192 | - | 0.320 | 0.073 |
| IL-20 | 0.359^(*)^ | 0.129 | 0.320 | - | 0.084 |
| CXCL9 | 0.313 | 0.410^*^ | 0.073 | 0.084 | - |
